# A systematic review on quality of life (QoL) of patients with peritoneal metastasis (PM) who underwent pressurized intraperitoneal aerosol chemotherapy (PIPAC)

**DOI:** 10.1515/pp-2021-0154

**Published:** 2022-04-21

**Authors:** Zhenyue Li, Louis Choon Kit Wong, Rehena Sultana, Hui Jun Lim, Joey Wee-Shan Tan, Qiu Xuan Tan, Jolene Si Min Wong, Claramae Shulyn Chia, Chin-Ann Johnny Ong

**Affiliations:** Department of Sarcoma, Peritoneal and Rare Tumours (SPRinT), Division of Surgery and Surgical Oncology, National Cancer Centre Singapore, Singapore, Singapore; Department of Sarcoma, Peritoneal and Rare Tumours (SPRinT), Division of Surgery and Surgical Oncology, Singapore General Hospital, Singapore, Singapore; Duke-NUS Medical School, Singapore, Singapore; Laboratory of Applied Human Genetics, Division of Medical Sciences, National Cancer Centre Singapore, Singapore, Singapore; SingHealth Duke-NUS Oncology Academic Clinical Program, Duke NUS Medical School, Singapore, Singapore; SingHealth Duke-NUS Surgery Academic Clinical Program, Duke NUS Medical School, Singapore, Singapore; Institute of Molecular and Cell Biology, A*STAR Research Entities, Singapore, Singapore

**Keywords:** meta-analysis, peritoneal metastasis, pressurized intraperitoneal aerosol chemotherapy, quality of life

## Abstract

**Background:**

Pressurized intraperitoneal aerosol chemotherapy (PIPAC) has recently emerged as a palliative alternative for patients with unresectable peritoneal metastasis (PM). Quality of life (QoL) has increasingly been used as an endpoint to evaluate treatment outcomes. This review aims to identify evidence on how PIPAC would impact the QoL of PM patients.

**Content:**

A systematic review was performed on articles identified from Medline, EMBASE, PsycInfo, and Web of Sciences. A meta-analysis was conducted on further selected studies. ACROBAT-NRSI was attempted to assess the risk of bias (RoB).

**Summary:**

Nine studies using the EORTC QLQ-C30 questionnaire to assess QoL after repeated PIPAC cycles were identified. Majority was found to be moderately biased and a great extent of heterogeneity was observed. Four studies on PM from either gastric cancer (GC) or epithelial ovarian cancer (EOC) were included for meta-analysis. In 31 GC patients and 104 EOC patients, QoL remained stable in 13/14 and 11/14 EORTC QLQ-C30 scales. PIPAC was inferior to cytoreductive surgery with hyperthermic intraperitoneal chemotherapy (CRS/HIPEC) in global QoL and functioning but superior in symptom reduction.

**Outlook:**

PIPAC is a well-tolerated option for most GC and EOC patients with irresectable PM. Future trials are warranted to confirm the findings.

## Introduction

Peritoneal metastasis (PM) refers to the formation of peritoneal tumors within the abdominal cavity. It is commonly observed in advanced stages of colorectal carcinoma (CRC), gastric carcinoma (GC), as well as epithelial ovarian carcinoma (EOC). Rarer causes of PM include primary peritoneal cancer, primary mesothelioma, and tumors arising from the small bowel, pancreas, and appendix [1]. Historically, PM was associated with a uniformly dismal prognosis due to delayed diagnosis, aggressive progression, and treatment resistance [1], [2], [3], [4], [5], [6]. Ascites and intestinal obstruction as late-stage symptoms are found in approximately 50% of patients with PM [5]. The various nonspecific complaints of PM patients during their initial presentations make early detection challenging. Surgery is potentially curative but patients are highly selected. Cytoreductive surgery with hyperthermic intraperitoneal chemotherapy (CRS/HIPEC) is the standard of care for PM patients with pseudomyxoma peritonei, peritoneal mesothelioma, and colorectal carcinoma with limited peritoneal spread [7]. More clinical trials are being conducted to investigate its efficacy in gastric and ovarian cancers. However, most patients are not eligible for CRS/HIPEC due to extensive disease not amenable for surgery, poor tumor biology, or lack of surgical fitness [7]. Systemic chemotherapy remains the standard of care for palliative patients but often show limited benefits due to drug resistance and poor distribution across the peritoneal–plasma barrier [2, 3, 8].

Recent advancements and the introduction of pressurized intraperitoneal aerosol chemotherapy (PIPAC) could potentially overcome the mechanisms of drug resistance and impaired bioavailability. PIPAC, performed laparoscopically, delivers a pressurized suspension of chemotherapeutic agents into the abdominal cavity via the use of a micropump and high-pressure injector. The rapid action and smaller dosage of therapeutic aerosols have been well-appreciated in pulmonary medicine [9]. Additionally, favorable aerosol deposition and lack of required maneuver coordination from patients make intraperitoneal administration easier [9]. Increased intra-abdominal pressure also facilitates intramural drug distribution [3]. PIPAC has therefore emerged as a promising palliative alternative for patients with PM [4]. Although PIPAC has been shown to improve outcome in selected patients in early phase studies, it remains unclear how the administration of PIPAC can improve the quality of life (QoL) of patients.

Biomedical data are traditionally evaluated as endpoints in clinical studies to identify treatment or intervention benefits. In the past decades, QoL research has increased worldwide to add onto biomedical endpoints for a more holistic assessment [10]. Self-reported QoL is important in understanding functioning, symptom relief, and rehabilitation of patients. It could aid medical professionals in treatment modification and improvement while prepare patients on potential consequences [10], [11], [12]. Here, we set out to evaluate the changes in QoL in PM patients who undergo PIPAC for palliation by undertaking a systematic review and meta-analysis.

## Methods

### Search strategy

**Figure 1:**
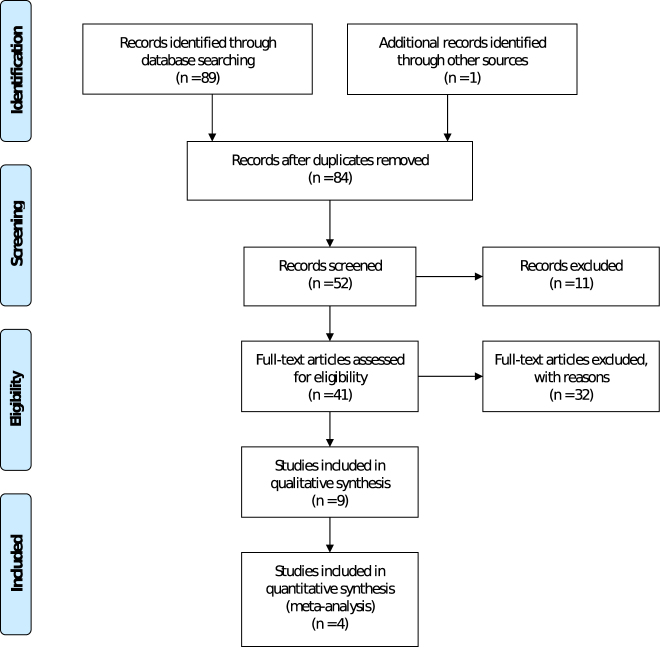
PRISMA flow diagram of the literature search algorithm.

A systematic literature search in Medline, EMBASE, PsycInfo, Web of Sciences, and Cochrane Central Register of Controlled Trials (CENTRAL) was conducted and analyzed according to the Preferred Reporting Items for Systematic Reviews and Meta-Analyses (PRISMA) guidelines [13]. Searches were performed independently by two reviewers (ZL and LCKW) for internal reliability. Our search strategy was generated based on the intervention and outcome components of the PICO formula [14] (Table 1). Studies in English, reporting on PIPAC together with its variations, and QoL together with its variations, were included. Our search was limited to the time period from 2011 to August 2020 before data extraction as the first in-human PIPAC was performed in 2011. Articles with full-text access were subjected to abstract screening and review articles were excluded. Full-text screening was subsequently performed to identify independent studies with available numerical or graphical questionnaire-based QoL scores. The reference lists of the excluded review articles were further scrutinized for potential unidentified research studies. Figure 1 illustrates the search flow.

**Table 1:** PICO [14] and search strategy.

**PICO**	

**Patients**	Patients with peritoneal metastasis (PM)
**Intervention**	Pressurized intraperitoneal aerosol chemotherapy (PIPAC)
**Comparative groups**	Trials having one treatment group consisting of patients with PM who underwent PIPAC plus or minus systemic chemotherapy, and one control group consisting of patients with PM who underwent only systemic chemotherapy
**Outcome**	Quality of life (QoL)

**Search strategy**	

**Intervention**	(PIPAC[All Fields] OR “Pressurized IntraPeritoneal Aerosol Chemotherapy”[All Fields] OR (“pressurized”[All Fields] AND “intraperitoneal”[All Fields] AND “aerosol”[All Fields] AND “chemotherapy”[All Fields]))

**AND**	

**Outcome**	((“quality of life”[MeSH Terms] OR (“quality”[All Fields] AND “life”[All Fields]) OR “quality of life”[All Fields]) OR QoL[All Fields] OR QLQ[All Fields] OR questionnaire[All Fields])

PM, peritoneal metastasis; PIPAC, pressurized intraperitoneal aerosol chemotherapy; QoL, quality of life.

### Meta-analysis

The meta-analysis was carried out with our best efforts to consolidate reported and unreported data. Data extractions mainly included: (1) PM histology, (2) number of PIPAC cycles, (3) intervals between PIPAC cycles, (4) number of patients who responded to the QLQ-C30 questionnaire for each PIPAC cycle, and finally (5) reported means and standard deviations of QoL. Most data can be found from the publications. The means of the QoL scores, with the exception of the GHS scale, of PM patients with EOC were approximated from the respective graphs. Corresponding authors of the included studies were contacted for PIPAC intervals and the number of patients in each PIPAC cycle.

PM from different primary sites is associated with distinctive symptoms, disease progression, and therapeutic chemotherapy drugs delivered through PIPAC. Thus, studies of single histology are clinically meaningful for analysis. Studies [15], [16], [17], [18] of single histology (GC or EOC) were identified for meta-analysis. EORTC QLQ-C30 assesses QoL in 30 questions from 15 scales – one global health status scale, five functioning scales, three multiple-item symptom scales, and six single-item symptom scales. Patient responses in each scale are then converted to a 0–100 range-based score. Due to a lack of data in the financial scale, our meta-analysis only incorporated 14/15 QLQ-C30 indices. In reference to how PIPAC affects QoL, we compared the QoL indices on the EORTC QLQ C30 questionnaire with data for 27 patients who underwent CRS/HIPEC [19].

Statistically, GC and EOC were repeatedly measured over time for each patient. With regard to time, patients with GC underwent PIPAC at week 8, 16, and 24, and patients with EOC underwent PIPAC at week 5, 10, 15, 20, 25, and 30. Differences between subgroups of GC or EOC patients at each timepoint were estimated using meta-regression based on repeated measures mixed-models (repeated ANOVA). This model accounts for the dependence among repeated measurements on the same patient. Mixed-models for continuous data was employed, in SAS v9.4 (SAS Institute, Cary, North Carolina; PROC MIXED), with time (in weeks), histology (GC or EOC), and time by histology interaction as fixed-effects (for example. GC at week 8), and time as a random-effect with a variance component variance–covariance matrix. The estimation method was based on a maximum likelihood technique and the variance–covariance matrix of the parameter estimates computed.

### Risk of bias

A risk of bias assessment based on QoL as the sole outcome was performed using the Cochrane Risk of Bias Assessment Tool: for Non-Randomized Studies of Interventions (ACROBAT-NRSI) tool [20]. Funnel plots were used to examine publication bias.

## Results

### Systematic search

A total of 29, 60, 0, 7, and 5 results were generated respectively from Medline, EMBASE, PsycInfo, Web of Sciences, and CENTRAL. After full-text review, eight studies were shortlisted [15], [16], [17], [18, 21], [22], [23], [24]. One relevant study was included from reference list search of previously excluded 21 review articles [25]. All shortlisted studies utilized the EORTC QLQ-C30 questionnaire. Homogeneity was achieved in all nine studies regarding the cohort study design, the PIPAC procedure, and QoL measurement. Heterogeneity was found regarding patient selection criteria, PM histology, number of PIPAC cycles, PIPAC administration interval, and EORTC QLQ-C30 administration intervals (Table 2).

**Table 2:** Details and characteristics of the relevant nine studies identified.

Publications	Year	n	Histology					Time of questionnaire administration	PIPAC interval	No. of responses per PIPAC cycle (c)	Representation of QoL scores
			GC	EOC	CRC	DMPM	Others				
Gockel et al. [15]^c^	2018	24	24					Before 3 PIPACs	8 weeks	Not reported	Mean ± SEM of 15 scales
Struller et al. [16]^c^	2019	14	14					Day 1 and after PIPAC 1	8 weeks	c1=c2=14	Mean ± SD of 15 scales
Tempfer et al. [17]^c^	2015	58^a^		84^a^		6	1	1 day before 8 PIPACs	4–6 weeks	c1=58, c2=31, c3=22, c4=9, c5=6, c6=4, c7=2, c8=1	Graph of mean ± 95% CI of 15 scales
Tempfer et al. [18]^c^	2015	46		46				1 day before 3 PIPACs	4–6 weeks	c1=46, c2=39, c3=28	Graph of mean ± 95% CI of 14 scales
Farinha et al. [22]^b^	2017	42	3	21	14	1	3	Before and after and follow up of 3 PIPACs	6 weeks	Not reported	Graph of 15 scales
Odendahl et al. [21]^b^	2015	48	18	8	8	1	13	Day 1 and after 2 PIPACs	6 weeks	c1=c2=c3=48	Graph of 9 scales
Graversen et al. [23]	2018	35	5	5	12	1	12	Day 1 and 60	60 days	Not reported	Graph of 1 scale
Giger–Pabst et al. [24]	2014	29				29		Not reported	6 weeks	c1=21, c2=18, c3=11, c4/5=11	Graph of 15 scales
Robella et al. [25]	2017	14	6	3	2	2	1	Not reported	6 weeks	Not reported	Not reported

	Total	310	70	167	36	40	30				

^a^The study was approximated as having single histology. 91 patients were recruited with specified histology but only 58 were interviewed with QLQ C-30. ^b^Studies with primary focus on quality of life. ^c^Studies selected for meta-analysis. GC, gastric cancer; EOC, epithelial ovarian cancer; CRC, colorectal cancer; DMPM, diffuse malignant peritoneal mesothelioma; PIPAC, pressurized intraperitoneal aerosol chemotherapy; QoL, quality of life.

### Meta-analysis

#### Global health status (GHS) scale

**Figure 2:**
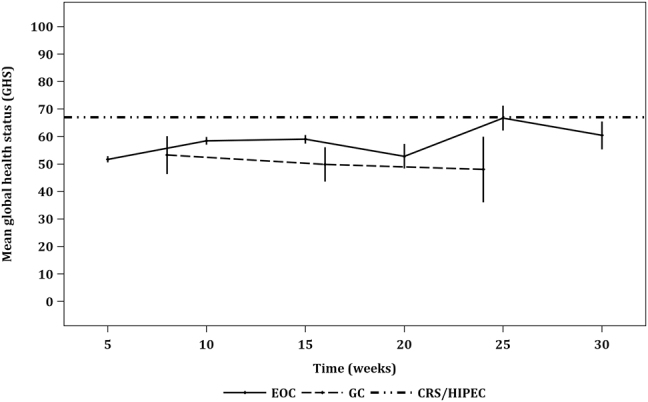
Line graph depicting the global health status (GHS) scale of QoL as measured by EORTC QLQ-C30 across time. The three-point trendline depicts GHS scores before the first, second, and third cycles of PIPAC (c) in patients with peritoneal metastasis (PM) from gastric cancer (GC); c1=31, c2=22, c3=5. The six-point trendline depicts GHS scores before the first, second, third, fourth, fifth, and sixth cycles of PIPAC in patients with PM from epithelial ovarian cancer (EOC); c1=104, c2=70, c3=50, c4=9, c5=6, c6=4. Error bars represent 95% CI. No significant difference was found comparing means within GC and EOC. As a reference, the dashed line represents a snapshot of GHS in 6–18 months after CRS/HIPEC in a cohort of patients (n=27) with PM from a mixed histological origin.

Calculated means ± 95% confidence intervals (CI) of the global health status (GHS) scale from GC patients, at weeks 8, 16, and 24, were 53.2 ± 6.9, 49.9 ± 6.3, and 48 ± 12, respectively. Calculated means ± 95% CIs of GHS from EOC patients at weeks 5, 10, 15, 20, 25, and 30 were 51.7 ± 1.2, 58.4 ± 1.4, 59 ± 2.5, 52.8 ± 4.5, 66.7 ± 5.6, and 60.4 ± 5.0, respectively (Figure 2). There was no significant difference in the QoL of GC patients who underwent PIPAC at the three reported timepoints. Overall, no deterioration of GHS was noted. Patients who underwent PIPAC had poorer GHS than patients who underwent CRS/HIPEC (Figure 2).

#### Functioning scales

**Figure 3:**
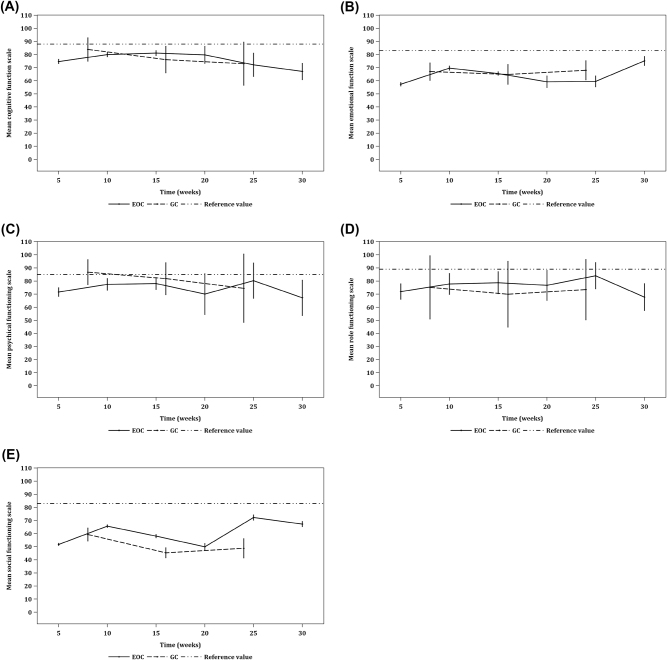
Line graphs depicting the five functioning scales of QoL as measured by EORTC QLQ-C30 across time. Line graphs of (A) cognitive functioning, (B) emotional functioning, (C) physical functioning, (D) role functioning, and (E) social functioning. Each three-point trendline depicts its respective functioning scores before the first, second, and third cycles of PIPAC (c) in patients with PM from gastric cancer (GC); c1=31, c2=22, c3=5. Each six-point trendline depicts its respective functioning scores before the first, second, third, fourth, fifth, and sixth cycles of PIPAC in patients with PM from epithelial ovarian cancer (EOC); c1=104, c2=70, c3=50, c4=9, c5=6, c6=4. Error bars represent 95% CIs. In each respective functioning scale of the five, no significant difference was found comparing means within GC and EOC. As a reference, each dashed line represents a snapshot of its respective functioning score in 6–18 months after CRS/HIPEC in a cohort of patients (n=27) with PM from a mixed histological origin.

Transient changes were detected in all five functioning scales, but overall, no deterioration in function in PM patients with either GC or EOC was noted. Patients who underwent PIPAC had poorer functioning than patients who underwent CRS/HIPEC (Figure 3).

#### Multiple-itemed symptom scales

**Figure 4:**
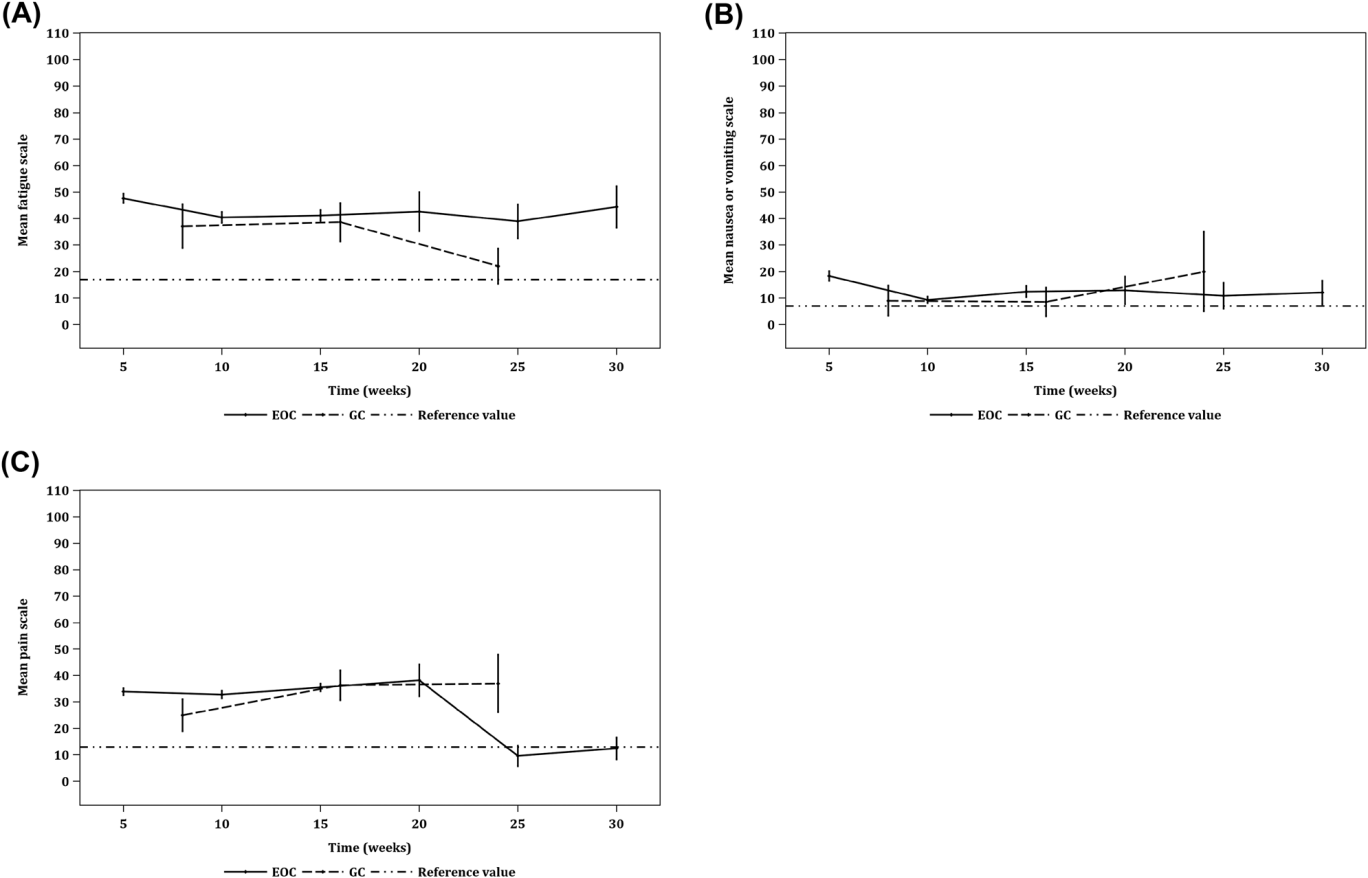
Line graphs depicting the three multiple-itemed symptom scales of QoL as measured by EORTC QLQ-C30 across time. Line graphs of (A) fatigue, (B) nausea and vomiting, and (C) pain. Each three-point trendline depicts its respective multiple-itemed symptom scores before the first, second, and third cycles of PIPAC (c) in patients with PM from gastric cancer (GC); c1=31, c2=22, c3=5. Each six-point trendline depicts its respective multiple-itemed symptom scores before the first, second, third, fourth, fifth, and sixth cycles of PIPAC in patients with PM from epithelial ovarian cancer (EOC); c1=104, c2=70, c3=50, c4=9, c5=6, c6=4. Error bars represent 95% CIs. Significant deterioration was found in the fatigue scale (FA) within GC and in the nausea and vomiting scale (NV) within EOC. As a reference, each dashed line represents a snapshot of its respective multiple-itemed symptom score in 6–18 months after CRS/HIPEC in a cohort of patients (n=27) with PM from a mixed histological origin.

In patients with GC, significant decrease in fatigue was noticed from baseline compared to before the third cycle of PIPAC, and similarly from before the second cycle of PIPAC to before the third cycle of PIPAC. This suggests progressive fatigue in patients with GC patients who undergo repeated PIPAC procedures. With regard to nausea or vomiting, significant decreases were found in patients with EOC from baseline to before the second, third, fourth, and fifth cycles of PIPAC, respectively, suggesting worsening nausea or vomiting in EOC patients with repeated PIPAC procedures. No deterioration of pain was noted in both GC and EOC patients. Moreover, patients who underwent PIPAC were found to have better multiple-itemed symptom reduction than patients who underwent CRS/HIPEC (Figure 4).

#### Single-itemed symptom scales

**Figure 5:**
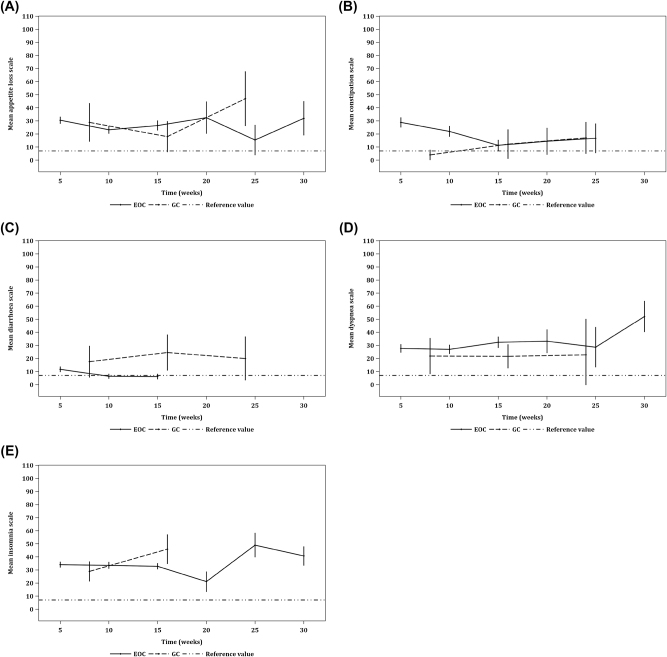
Line graphs depicting the single-itemed symptom scales of QoL as measured by EORTC QLQ-C30 across time. Line graphs of (A) appetite loss, (B) constipation, (C) diarrhea, (D) dyspnea, and (E) insomnia. Each three-point trendline depicts its respective single-itemed symptom scores before the first, second, and third cycles of PIPAC (c) in patients with PM from gastric cancer (GC); c1=31, c2=22, c3=5. Each six-point trendline depicts its respective single-itemed symptom scores before the first, second, third, fourth, fifth, and sixth cycles of PIPAC in patients with PM from epithelial ovarian cancer (EOC); c1=104, c2=70, c3=50, c4=9, c5=6, c6=4. The diarrhea scale (DI) for EOC patients only has three data points before the first, second, and third cycles of PIPAC whereas the constipation scale (CO) for EOC patients only has five data points from the first, second, third, fourth, and fifth cycles of PIPAC. Error bars represent 95% CIs. Significant deterioration was found in DI and CO within EOC. As a reference, each dashed line represents a snapshot of its respective single-itemed symptom score in 6–18 months after CRS/HIPEC in a cohort of patients (n=27) with PM from a mixed histological origin.

Scattered significant increases of dyspnea were identified. Despite data of fewer PIPAC cycles collected for analysis, patients with EOC were found to have worsening diarrhoea from baseline to the second cycle of PIPAC, and from baseline to the third cycle of PIPAC. Transient changes were picked up in appetite loss and insomnia, but overall our analysis showed stabilization in patients with GC and EOC. Statistically significant aggravating constipation was detected in EOC patients from baseline to all subsequent timepoints respectively. Patients who underwent PIPAC were found to have better single-itemed symptom reduction than patients who underwent CRS/HIPEC (Figure 5).

### Risk of bias

Table 3 summarizes the risk of bias in selected studies as determined by the ACROBAT-NRSI tool [20]. Seven studies [15], [16], [17], [18, 21, 22, 24] were determined to have moderate risks. Graversen et al. [23] was subject to serious risk of bias and Robella et al. [25] lacks essential information. Specifically, time-varying bias introduced from having lesser patients eligible for subsequent cycles of PIPAC was identified in seven studies [15, 17, 18, 22], [23], [24], [25]. Selection bias was more profound in Gockel et al. [15] that expands treatment to ineligible patients with strong preferences and Odendahl et al. [21] that recruits patients resistant to systemic chemotherapy. PIPAC intervention was considered similar across all institutions and the EORTC QLQ-30 was validated and universally applied. As for departures from PIPAC, Gockel et al. [15], Graversen et al. [23], and Giger–Pabst et al. [24] were subject to bias induced by systemic chemotherapy as co-intervention. Missing data are likely to introduce bias because patients lasted longer for repeated cycles of PIPAC and patients who responded to QLQ-C30 tend to have better QoL. In almost all studies, fewer patients were interviewed with QLQ-C30 than those who were recruited. Patients who responded to QLQ-C30 were even fewer than the ones interviewed, which subject those studies to biases caused by missing data. Finally, studies that reported some scales in QLQ-C30 but not others were suspected of selective outcome and analysis reporting bias. Giger–Pabst et al. [24] reported only GHS, which may contribute to analysis reporting bias. Overall, the pooled analysis from four studies [15], [16], [17], [18] for meta-regression was considered to have moderate risk of bias. No significant publication bias could be observed as determined by the funnel plots (Supplementary Figures S1 and S2).

**Table 3:** Risk of bias (RoB) using ACROBAT-NRSI tool [20]. Overall RoB for each study is considered (1) low if the study is judged to be at low RoB for all domains, (2) moderate if the highest risk among all domains is moderate (3) serious if the highest risk among all domains is serious, or (4) critical if the highest risk among all domains is critical [46].

Publications	Year	Bias due to confounding	Bias in selection of participants into the study	Bias in measurement of interventions	Bias due to departures from intended interventions	Bias due to missing data	Bias in measurement of outcomes	Bias in selection of the reported results	Overall bias
Gockel et al. [15]^b^	2018	Moderate	Moderate	Low	Moderate	Moderate	Low	Low	Moderate
Struller et al. [16]^b^	2019	Low	Low	Low	Low	Moderate	Low	Low	Moderate
Tempfer et al. [17]^b^	2015	Moderate	Low	Low	Low	Moderate	Low	Moderate	Moderate
Tempfer et al. [18]^b^	2015	Moderate	Low	Low	Low	Moderate	Low	Low	Moderate
Farinha et al. [22]^a^	2017	Low	Low	Low	Low	Low	Low	Low	Moderate
Odendahl et al. [21]^a^	2015	Low	Moderate	Low	Low	Moderate	Low	Moderate	Moderate
Graversen et al. [23]	2018	Moderate	Low	Low	Moderate	Unclear	Low	Serious	Serious
Giger–Pabst et al. [24]	2014	Moderate	Low	Low	Moderate	Moderate	Low	Low	Moderate
Robella et al. [25]	2017	Moderate	Low	Low	Low	Unclear	Low	Unclear	Unclear

^a^Studies with primary focus on quality of life (QoL). ^b^Studies selected for meta-analysis.

## Discussion

All nine studies have the same cohort study design, PIPAC intervention, and EORTC QLQ-C30 questionnaire used for QoL assessment (Table 2). They nonetheless vary in patient selection criteria, primary tumor origin, number of PIPAC cycles, PIPAC administration interval, and EORTC QLQ-C30 administration time (Table 2). Specifically, Struller et al. [16] and Tempfer et al. [17] are the only studies that provided calculated minimum sample size to guide recruitment. Odendahl et al. [21] and Farinha et al. [22] are the only studies with primary aims in QoL. Other studies that focused on tumor regression, postoperative complications, or median or overall survival, have underreported QoL data. Limitations commonly discussed in the nine studies were (1) small sample size, (2) nonrandomized design, and (3) patients pretreatment heterogeneity. Limitations commonly discussed in the four studies we extracted for the meta-analysis include: (1) small numbers of PIPAC cycles, (2) fewer and fewer patients suitable for repeated PIPAC cycles, and (3) not full return of the QLQ-C30 questionnaire. Moreover, the self-selection of patients undergoing PIPAC may play an important role with respect to their QoL. Patients who report higher QoL are more likely to continue with repeated PIPAC cycles. Contrastingly, patients with lower QoL or suffer from side effects following treatment with PIPAC may elect to stop their treatment. This translates to a patient selection bias where only highly selected patients are enrolled into subsequent PIPAC cycles, possibly inflating the positive QoL results of PIPAC. Accordingly, future studies should adequately address the influence of this confounding factor.

The pooled analysis performed in this study provided us with greater power to detect for differences between groups. No QoL deterioration was found in 13 of 14 scales of patients with GC and no QoL deterioration was found in 11 of 14 scales of patients with EOC. This is consistent with the conclusion drawn from the nine articles that PIPAC is well-tolerated in most patients with PM. Furthermore, the observed differences between the GC and EOC subgroups provide evidence that the histological origin has an influence on the QoL of patients as well as patient outcome, particularly in the social functioning and fatigue scales of QoL. Down-trending of QoL curves across time, on the other hand, might not be contributed entirely to PIPAC, but to the natural aggregating progression of PM. One possible explanation is that without PIPAC, patients might experience even worse QoL. To put our results into perspective, it would have been ideal to compare the QoL of PIPAC patients with PM patients undergoing other palliative cancer therapies, such as systemic chemotherapy, of corresponding histological origins. Unfortunately, there is a paucity of evidence investigating the QoL of patients with advanced PM who have undergone palliative treatment to control their cancer symptoms. Notwithstanding, PIPAC has shown efficacy in clinical survival and tumor regression in patients with PM of CRC, GC, and EOC origins with reported resistance to systemic chemotherapy [18, 26, 27]. This suggests that PIPAC treatment could potentially lead to better QoL in patients as compared to other palliative treatment options such as systemic chemotherapy.

To this end, we compared the QoL of PIPAC patients with 27 patients who underwent CRS/HIPEC. Acknowledging the multiple limitations such as differences in patients’ disease stage, it is our opinion that the comparison remains helpful in contextualizing the relative degree of QoL improvement in PIPAC patients. We found that patients who underwent PIPAC showed lower QoL compared to CRS/HIPEC in the GHS scale and five functioning scales. This is consistent with our understanding that patients selected for a curative modality would have better general and functioning status than palliative patients. Interestingly, patients who underwent PIPAC showed better QoL in the nine symptom scales than CRS/HIPEC, suggesting that PIPAC is a promising palliative treatment for patients with PM who are not eligible for CRS/HIPEC. Another likely explanation could be that data were never collected or extracted long enough postsurgically for CRS/HIPEC to show its superiority over PIPAC. Conclusively, few existing studies have examined the impact of PIPAC on QoL. As the power of this meta-analysis is limited by study numbers and sample size, we would like to call in the future for more carefully conducted two-armed randomized controlled trials in patients with PM from a single origin to confirm our findings. Ideally, a larger number of PM patients with the same histological origin should be recruited under standardized institutional guidelines regarding pretreatment, withdrawal, PIPAC interval, and QoL questionnaire administration and collection. Half of the patients should be randomly selected to receive PIPAC alone or in combination with systemic chemotherapy while the other half should receive systemic chemotherapy as the control group.

The widespread heterogeneity in these nine included studies, namely patient pretreatment, PM histology, PIPAC interval, and QLQ-C30 interval, as well as the inherent moderate risk of bias, has undermined our meta-analysis to some extent. To overcome this limitation, we performed a pooled analysis to increase our sample size and subcategorize PM by GC or EOC histology. The pooled analysis granted us higher power to detect for differences. Furthermore, despite our best efforts to contact corresponding authors, data for patients with EOC were only close estimation because the published work did not report the exact QoL numerical score values. As such, we approximated the data from the published figures. Furthermore, as far as we know, there is currently no standardized quality assessment for nonrandomized studies. In order to evaluate the risk of bias in our selected studies, we utilized Cochrane’s ACROBAT-NRSI tool as it assesses the studies’ internal validity. To our knowledge, the ACROBAT-NSRI is commonly used in other systematic reviews [28], [29], [30], [31], [32], [33], [34], [35], [36], [37], [38], [39], [40], [41], [42], [43], [44], [45] and hence we have adopted the same. Additionally, CRS/HIPEC and PIPAC are procedures for distinct indications. The former is a single curative procedure whereas the latter involves multiple cycles of palliative treatment. Direct comparison of patient QoL for these two procedures would present with challenges, such as identifying the appropriate timepoint after surgery for data analysis. We will be looking into more rigorous evaluation of QoL in subsequent studies.

## Conclusions

PIPAC is a well-tolerated option for most GC and EOC patients with irresectable PM. Compared to CRS/HIPEC, PIPAC provided better stabilization of QoL in symptom reduction. PIPAC could be a viable option to palliate symptoms with irresectable PM. More carefully conducted randomized studies are needed in this area to validate our findings.

## Supplementary Material

Supplementary Material
