# Supplementary material for: A systematic review on quality of life (QoL) of patients with peritoneal metastasis (PM) who underwent pressurized intraperitoneal aerosol chemotherapy (PIPAC)
Source: Pleura Peritoneum. 2022 Apr 21;7(2):39–49. doi: 10.1515/pp-2021-0154 (PMC9166188; doi:10.1515/pp-2021-0154)
Supplement: Supplementary file 1 — Supplementary Material [file j_pp-2021-0154_suppl.docx]

**A systematic review on quality of life (QoL) of patients with peritoneal metastasis (PM) who underwent pressurized intraperitoneal aerosol chemotherapy (PIPAC)**

Zhenyue Li^a,b,c,#^, Louis Choon Kit Wong^a,b,c,#^, Rehena Sultana^c^, Hui Jun Lim^a,b,d^, Joey Wee-Shan Tan^a,b,d^, Qiu Xuan Tan^a,b,d^, Jolene Si Min Wong^a,b,e,f^, Claramae Shulyn Chia^a,b,e,f^, Chin-Ann Johnny Ong^a,b,d,e,f,g^*,

^a^ Department of Sarcoma, Peritoneal and Rare Tumours (SPRinT), Division of Surgery and Surgical Oncology, National Cancer Centre Singapore, Singapore

^b^ Department of Sarcoma, Peritoneal and Rare Tumours (SPRinT), Division of Surgery and Surgical Oncology, Singapore General Hospital, Singapore

^c^ Duke-NUS Medical School, Singapore

^d^ Laboratory of Applied Human Genetics, Division of Medical Sciences, National Cancer Centre Singapore, Singapore

^e^ SingHealth Duke-NUS Oncology Academic Clinical Program, Duke NUS Medical School, Singapore

^f^ SingHealth Duke-NUS Surgery Academic Clinical Program, Duke NUS Medical School, Singapore

^g^ Institute of Molecular and Cell Biology, A*STAR Research Entities, Singapore

^#^ Equal contribution

* Corresponding author

**Corresponding author:**

Assistant Professor Chin-Ann Johnny Ong

Department of Sarcoma, Peritoneal and Rare Tumours (SPRinT), Division of Surgery and Surgical Oncology, National Cancer Centre Singapore

Address: 11 Hospital Crescent, Singapore 169610

Tel no.: (65) 6436 8318 Fax no.: (65) 6225 7559 Email: johnny.ong.c.a@singhealth.com.sg

**Word count: 2849**

**Number of tables and figures: 8**

**Supplementary material: Yes**

**Supplementary Figure S1** Symmetrical funnel plots of global health status (GHS) and functioning scales in the selected quality of life (QoL) studies indicate absence of significant publication bias.

Funnel plots of (A) GHS, (B) cognitive functioning, (C) emotional functioning, (D) physical functioning, (E) role functioning, and (F) social functioning.

**Supplementary Figure S2** Symmetrical funnel plots of symptom scales in the selected quality of life (QoL) studies indicate absence of significant publication bias.

Funnel plots of (A) appetite loss, (B) constipation, (C) diarrhoea, (D) dyspnoea, (E) insomnia, (F) fatigue, (G) nausea and vomiting, and (H) pain.
